# Traditional and Emerging Strategies for Managing Polymyalgia Rheumatica: Insights into New Treatments

**DOI:** 10.3390/jcm13216492

**Published:** 2024-10-29

**Authors:** Carlos García-Porrúa, Elena Heras-Recuero, Teresa Blázquez-Sánchez, Arantxa Torres-Roselló, Santos Castañeda, Miguel Ángel González-Gay

**Affiliations:** 1 Division of Rheumatology, Hospital Lucus-Augusti, 27003 Lugo, Spain; carlos.garcia.porrua@sergas.es; 2 Division of Rheumatology, IIS-Fundación Jiménez Díaz, 28040 Madrid, Spain; elena.herasr@fjd.es (E.H.-R.); teresa.blazquezs@quironsalud.es (T.B.-S.); arantxa.torres@quironsalud.es (A.T.-R.); 3 Division of Rheumatology, Hospital Universitario de la Princesa, Instituto de Investigación del Hospital de La Princesa, 28006 Madrid, Spain; scastas@gmail.com; 4 Medicine and Psychiatry Department, University of Cantabria, 39008 Santander, Spain

**Keywords:** polymyalgia rheumatica, relapses, prednisone, methotrexate, leflunomide, biologic agents, tocilizumab, sarilumab, JAK inhibitors

## Abstract

**Background/Objectives:** Polymyalgia Rheumatica (PMR) is an inflammatory condition that primarily affects individuals aged 50 and older, especially in Western countries. Although glucocorticoids are the cornerstone of PMR treatment, these drugs are associated with side effects, making it advisable to use them for the shortest duration possible. However, tapering or discontinuation of glucocorticoids often leads to disease relapses. In this review, we focus on the traditional management of PMR, as well as the potential for therapies that may reduce glucocorticoid use. Special attention is given to the efficacy of biologic agents in PMR management. **Methods:** A literature review, primarily based on articles published in PubMed, was conducted. In addition to discussing various glucocorticoids and conventional disease-modifying drugs used for the management of isolated PMR, this review specifically focused on the information reported regarding new therapies, with particular emphasis on biologic agents. **Results:** Prednisone or prednisolone at a dose ranging between 12.5 and 25 mg/day is the agreed-upon treatment for PMR. Due to the side effects associated with prolonged glucocorticoid use and the high frequency of relapses when glucocorticoids are tapered, glucocorticoid-sparing agents have emerged as tools in the management of PMR. Methotrexate has traditionally been the conventional disease-modifying antirheumatic drug (DMARD) unanimously recommended for use in PMR. Other conventional DMARDs, such as leflunomide, have shown promising results but require further study. The use of biologic agents has marked a significant step forward in the management of PMR. While anti-TNF agents failed to provide beneficial effects in isolated PMR, anti-IL-6 receptor agents, such as tocilizumab and sarilumab, have demonstrated efficacy in reducing relapse frequency, lowering the cumulative glucocorticoid burden, and achieving long-term remission of the disease. Other biologic agents, many of which have been used in giant cell arteritis, as well as Janus kinase (JAK) inhibitors, are currently under investigation. **Conclusions:** Glucocorticoids are the primary treatment for isolated PMR but are associated with comorbidities, especially in patients with pre-existing conditions or frequent relapses. Glucocorticoid-sparing agents, such as methotrexate and biologics, in particular tocilizumab and sarilumab, offer alternatives, improving symptoms and reducing glucocorticoid use. While biologic agents reduce long-term side effects and help achieve disease remission, their use must consider potential side effects and higher costs compared to traditional therapies.

## 1. Introduction

Polymyalgia Rheumatica (PMR) is an inflammatory condition characterized by muscle pain and stiffness that primarily affects the shoulders, upper arms, neck, hips, and thighs. PMR patients experience severe symmetrical muscle stiffness, particularly in the morning or after periods of inactivity. In addition to general fatigue, symptoms can include fever, generally low grade, weight loss, and sometimes mild swelling in the hands or feet [1,2].

PMR primarily affects adults over 50, occurring more frequently in women than in men, and is more common among individuals of Northern European ancestry. Its incidence follows a north–south gradient, being higher in Scandinavian countries and lower in Southern European populations [3,4,5].

Most patients with PMR have elevated markers of inflammation in blood tests. However, a small proportion may exhibit mildly increased or normal values [6].

The 2012 ACR/EULAR criteria for PMR indicate that patients aged 50 years and older with bilateral shoulder aching and abnormal CRP and/or ESRs can be classified as having PMR if they also experience morning stiffness lasting more than 45 min, along with new hip involvement (pain, tenderness, or restricted movement). The likelihood of PMR is further increased by the absence of peripheral synovitis or positive rheumatoid arthritis (RA) serology. Although a rheumatoid factor can occasionally be present in PMR patients, its absence is a valuable criterion for distinguishing PMR from RA in older adults [7].

Genetic predisposition contributes to the development of PMR [8], with certain human leukocyte antigen (HLA) types being linked to a higher risk [9]. Additionally, gene polymorphisms outside the HLA region have also been associated with this condition [10,11]. Environmental factors, such as infections, may trigger the onset of PMR in individuals with genetic susceptibility [4,6]. However, the precise nature of these triggers is still unclear. Some research indicates seasonal variations in PMR incidence, with a higher number of cases diagnosed during winter, possibly due to environmental factors like infections. There have been reports of PMR occurring following influenza B and, more recently, the SARS-CoV-2 infection [12,13].

The exact cause of PMR is still unknown, but it involves inflammation of the synovial membranes in joints, bursae, and surrounding structures like tendons, leading to pain and stiffness. Arthroscopic exams show mild synovitis in proximal joints, primarily involving macrophages and CD4 T-cells, though this does not fully explain the widespread pain seen in PMR. Imaging techniques like magnetic resonance imaging (MRI) and ultrasonography (US) have revealed conditions like subacromial/subdeltoid bursitis and biceps tenosynovitis, indicating that extra-articular structures are also affected. PMR can also cause hip effusions, pelvic bursitis, and less commonly, trochanteric and iliopsoas bursitis. Ischiogluteal bursitis, which is relatively specific in PMR, and interspinous bursitis in the cervical and lumbar spine, might explain the associated neck and back pain [14].

The 2012 EULAR/ACR criteria for PMR include US data, highlighting the importance of bilateral subacromial/subdeltoid and trochanteric bursitis in diagnosing PMR [7]. Ultrasound can improve diagnostic accuracy for PMR by identifying bilateral shoulder abnormalities (e.g., subacromial bursitis or glenohumeral effusion) or a combination of shoulder and hip issues (e.g., hip effusion) [7]. PET-CT is also helpful [15,16]. The diagnosis remains largely clinical, requiring the exclusion of other conditions like late-onset RA, spondyloarthritis, or systemic lupus erythematosus [17,18,19,20].

Subclinical GCA in patients with PMR refers to the presence of large-vessel inflammation that is not accompanied by the typical cranial symptoms of GCA, such as headache or jaw claudication [21]. Studies using imaging techniques like FDG-PET-CT and ultrasound have identified evidence of inflammation in large vessels, such as the aorta and its branches, in up to one-third of patients with isolated PMR. This suggests that some PMR patients might have underlying GCA, even in the absence of classic symptoms [1,22]. This subclinical form of GCA is important to recognize because it may increase the risk of complications like aortic aneurysms or dissections if left untreated. The identification of subclinical GCA through advanced imaging highlights the need for careful monitoring of PMR patients, particularly those with systemic symptoms or poor response to glucocorticoid therapy [21,23,24,25].

## 2. Relapses: A Common Problem in the Management of PMR

Relapses occur in 20% to 60% of patients, mainly during the first year of treatment [26,27,28], and are characterized by the return of symptoms similar to those at diagnosis, typically presenting as pain and stiffness in the shoulders and arms [29,30]. Relapses often correlate with increased inflammatory markers, such as the elevated erythrocyte sedimentation rate (ESR) and C-reactive protein (CRP). Persistent high levels of CRP and interleukin (IL)-6 have been identified as predictors for relapses in PMR patients [31]. CRP is now regarded as a more reliable marker for monitoring inflammation in PMR compared to the ESR [32,33,34]. However, in patients experiencing relapse, about 25% may have normal ESR values, and 15% might have normal CRP levels [35,36,37].

Relapses, along with the concepts of remission and disease activity, have been defined inconsistently across clinical studies [38]. The tools used to assess these disease situations require further validation. Additionally, qualitative research is necessary to gain a deeper understanding of remission and relapses in PMR. In this regard, properly identifying relapses is crucial for adjusting glucocorticoid treatment effectively. The PMR activity score (PMR-AS) has been proposed as a promising useful tool to define relapses and guide therapy adjustments [29,39,40]. However, evidence supporting its use for measuring remission and relapses remains limited [38].

Risk factors for relapses are reported with inconsistent results across studies. In general, relapses of PMR generally occur when the glucocorticoid dose is tapered [41]. For this reason, in patients with isolated PMR, the primary factor influencing the relapse risk is the speed of glucocorticoid tapering [42,43,44]. A slower reduction in prednisone (less than 1 mg/month after starting at 15 mg/day) tends to lower the risk of relapse, while a more rapid taper increases this risk [42,43,45,46]. However, research involving a Korean patient cohort suggested that the rate of glucocorticoid tapering did not correlate with relapse rates, implying a possible genetic influence [47]. Genetic studies have shown that certain HLA-DRB1 alleles, particularly the rheumatoid arthritis (RA)-shared epitope, are associated with a higher likelihood of relapses [48]. In PMR patients from Northwest Spain, individuals with the RA-shared epitope, especially those with the HLA-DRB1*0401 allele, experienced more frequent relapses [43]. Additionally, genetic polymorphisms leading to continuous IL-6 elevation can further increase the relapse risk [49].

Current guidelines for managing PMR relapses are based primarily on an expert consensus and retrospective analyses. Classic management involves resuming or adjusting glucocorticoid therapy [1]. According to the British Society of Rheumatology, the recommended starting dose of prednisolone is 15 mg for three weeks, followed by a gradual tapering process [50]. Generally, glucocorticoid treatment lasts between 12 and 24 months, with therapy exceeding two years necessitating further investigation for other potential underlying conditions.

For managing relapses specifically in isolated PMR, the EULAR-ACR guidelines recommend increasing the prednisone dose to the previous level, followed by a gradual taper back to the relapse dose within 4–8 weeks [51]. In some cases, an increase of 2.5 to 5 mg/day may be needed to control symptoms [43]. Patients experiencing more than two relapses may require the addition of other immunosuppressive therapies.

## 3. Treatment of PMR

A recent systematic literature review assessed the current evidence on Treat-to-Target (T2T) strategies for PMR and GCA. The review included studies from Medline, EMBASE, the Cochrane Library, Clinicaltrials.gov, and the EULAR/ACR abstract database through May 2022 [52]. Of 7809 abstracts screened, 76 studies, including 31 randomized clinical trials, met the criteria for inclusion. However, the review did not identify any studies that directly compared a T2T strategy with standard care. Most PMR trials focused on treatment outcomes, particularly cumulative glucocorticoid doses and taper, along with clinical, laboratory, and safety outcomes. These findings not only provide evidence on current T2T strategies but also highlight important knowledge gaps, offering a basis for developing future T2T recommendations for PMR and GCA [52].

Below, we describe the different therapies used for the management of PMR.

### 3.1. Glucocorticoids: The Mainstay of PMR Treatment

The primary goal in treating PMR is to control symptoms and prevent relapses, with oral prednisone/prednisolone being the main treatment [53,54,55]. The recommended starting dose is 12.5–25 mg/day, individualized based on patient factors [54,56]. For those with conditions like diabetes or osteoporosis, a lower dose of 12.5–15 mg/day is advised. A single daily dose of glucocorticoids is generally recommended [54,56], though a divided dose can sometimes provide quicker relief for severe symptoms [1]. Symptom improvement usually occurs within the first week, often within 72 h, with normalization of the ESR and CRP within 2–4 weeks [55,56,57,58].

Optimal methods for glucocorticoids tapering are still under research, with most guidelines based on expert opinion. Typically, an initial prednisone dose is maintained for 3–4 weeks before gradual tapering. For example, starting at 15 mg/day, the dose is reduced to 12.5 mg daily for 2–4 weeks, then 10 mg daily for 4–6 weeks, followed by monthly reductions of 1–1.25 mg or every 2–3 months by 2.5 mg [1,59,60].

The circadian rhythm of symptoms in patients with chronic inflammatory diseases such as PMR is well known [61]. Based on that, additional innovation may involve modified-release prednisone, which allows for the timed release of the drug to align with the body’s inflammatory cycle. Administered at bedtime, the drug releases at around 2 a.m., optimizing the suppression of proinflammatory cytokines that peak in the early morning [62]. This approach has shown positive results in GCA when compared to immediate-release prednisone, suggesting its potential benefits for PMR as well. With respect to this, Cutolo et al. assessed the efficacy and safety of modified-release prednisone compared to immediate-release prednisone in newly diagnosed glucocorticoid-naïve patients with PMR [63]. For this purpose, these investigators conducted a double-blind, randomized trial involving sixty-two patients with PMR, who received either modified-release prednisone (15 mg/day taken at approximately 22:00) or immediate-release prednisone (15 mg/day taken in the morning) for 4 weeks. The primary endpoint was the complete response rate, defined as at least a 70% reduction in the PMR visual analogue scale, duration of morning stiffness, and CRP levels (or CRP < 2 × the upper limit of normal) at week 4. They found that the complete response rate at week 4 was higher for modified-release prednisone (53.8%) compared to immediate-release prednisone (40.9%) [63]. Therefore, this study indicated a favorable trend for modified-release prednisone when compared with immediate-release prednisone, suggesting that further research is warranted.

Injections, like 6-methylprednisolone or intramuscular methylprednisolone acetate, have limited efficacy [64,65]. Non-steroidal anti-inflammatory drugs (NSAIDs) are not recommended due to limited benefits and potential complications.

### 3.2. Glucocorticoid-Sparing Agents

In patients with PMR who experience severe glucocorticoid-related side effects, it is often necessary to use conventional immunosuppressive drugs or biologic agents as glucocorticoid-sparing alternatives to manage the condition. These agents are particularly useful not only in cases where patients develop significant side effects from glucocorticoid therapy (such as osteoporosis, diabetes, or hypertension), but also in patients who require prolonged glucocorticoid therapy due to refractory disease or, more commonly, because of relapsing disease.

#### 3.2.1. Conventional Glucocorticoid-Sparing Agents

Conventional disease-modifying anti-rheumatic drugs have been used in patients with PMR as glucocorticoid-sparing agents. The use of these drugs aimed to reduce glucocorticoid-related side effects or manage refractory/relapsing cases. Among them, methotrexate has been the most commonly used conventional disease-modifying anti-rheumatic drug in PMR, typically starting at 10 to 15 mg per week. In this regard, Caporalli et al. carried out a randomized, double-blind, placebo-controlled trial that included 72 newly diagnosed patients with PMR who were treated with either 10 mg of oral methotrexate or a placebo, in addition to prednisone at an initial dose of starting at 25 mg/day and alongside a tapering course of prednisone to 0 mg/day over a period of 24 weeks, with adjustments for relapses. This study showed a significant reduction in the need for glucocorticoids following methotrexate therapy. In this regard, at 76 weeks, the median prednisone dose was 2.1 g in the methotrexate group compared with 2.97 g in the placebo group [66]. Also, Ferraccioli et al. conducted another noteworthy study aimed at evaluating the benefits of intramuscular MTX in PMR patients [67]. In this one-year prospective study, 24 newly diagnosed PMR patients were randomized to receive either 10 mg/week of intramuscular MTX plus prednisone or prednisone alone. At 12 months, all patients had achieved clinical remission, with both groups showing normal levels of acute phase reactants [67]. Notably, the cumulative prednisone dose by 12 months was significantly lower in the MTX group compared to the placebo group. Additionally, bone mineral density significantly declined in the placebo group but remained stable in the MTX group [67]. These findings suggest that MTX may offer a glucocorticoid-sparing effect and help reduce the risk of osteoporosis in PMR patients.

Based on this evidence, the 2015 EULAR/ACR guidelines recommend considering early methotrexate use for patients at high risk of relapses or those facing prolonged therapy, especially if they have comorbidities or are on other medications that increase the likelihood of glucocorticoid side effects [56]. However, Van der Veen et al. conducted a randomized, double-blind, placebo-controlled trial that yielded negative results. The study involved 40 patients with PMR, including six with GCA, who were given either 7.5 mg of oral methotrexate or a placebo, along with a tapering course of glucocorticoids starting with 20 mg/day of prednisone. The trial did not demonstrate a significant glucocorticoid-sparing effect of methotrexate. Specifically, there were no notable differences between the methotrexate and placebo groups with respect to time to achieve remission, duration of remission, number of relapses, or total doses of prednisone administered [68].

The use of leflunomide has also been assessed in a few studies. In this regard, Diamantopoulos et al. conducted a retrospective assessment of eleven GCA and twelve PMR difficult-to-treat patients. They began treatment with 10 mg/day of leflunomide, which could be increased to 20 mg if the clinical response was inadequate or at the discretion of the treating physician. Six patients (26%)—three with PMR and three with GCA—discontinued treatment due to side effects, although no serious adverse events requiring hospitalization were reported. Additionally, five of the 23 patients (two with PMR and three with GCA) stopped treatment after achieving remission, with a mean duration of 10.2 months. In the PMR group, there was a 6 mg/dl reduction in CRP levels and a 34.2% decrease in the prednisolone dose [69].

In a separate study, Adizie et al. assessed the efficacy and side effects of leflunomide in nine patients with GCA and fourteen with PMR [70]. All of them had problems to taper their prednisolone doses, and three had not responded to optimal doses of methotrexate. A starting dose of 10 mg/day of leflunomide was administered to patients three to nine months after the initiation of glucocorticoid therapy. The leflunomide dose was adjusted to either 10/20 mg on alternate days for five patients or 20 mg/day for two patients, depending on clinical and biochemical responses. Overall, leflunomide was well tolerated by all but three patients. All patients with GCA and all but one in the isolated PMR group showed a complete or partial response to leflunomide. Glucocorticoid therapy was discontinued in nine patients and tapered in twelve of twenty-three patients with GCA or PMR. Although the study was open label, without a randomized design, and lacked a control group, the results indicated that leflunomide is well tolerated in patients with GCA and PMR, which might aid glucocorticoid tapering.

A recent comparison of leflunomide and methotrexate in PMR patients indicated that leflunomide might be more effective in facilitating glucocorticoid tapering [71]. In this regard, Vinicki et al. evaluated one hundred and forty-three patients with PMR treated with methotrexate (median dose 15 mg/week) and forty-three patients who received a fixed dose of leflunomide (20 mg/day). The glucocorticoid doses at baseline and during tapering were determined by the treating physician and were not pre-established. The sampling of centers was not randomized, and patients underwent a follow-up period of at least three months from the start of the conventional disease-modifying drugs. Withdrawal from glucocorticoids was more common in the leflunomide group (72%) compared to the methotrexate group (39%). With respect to this, the time to prednisone discontinuation was shorter for patients treated with leflunomide, with a median of 4.7 months compared to 31.8 months for those on methotrexate. Additionally, a multivariate analysis indicated a significantly higher probability of remission associated with leflunomide therapy [71]. However, the study lacked long-term follow-up, particularly for the leflunomide group. Therefore, we believe that prospective studies involving PMR patients are necessary to further validate these findings regarding the efficacy of leflunomide in this condition [72].

Methotrexate and leflunomide are currently being investigated in phase III randomized, double-blind, placebo-controlled trials for patients newly diagnosed with PMR [73,74]. One such trial is the PolyMyalgia Rheumatica treatment with Methotrexate in Optimal Dose in an Early Disease Phase (PMR MODE) study. This study is designed to evaluate the efficacy of methotrexate at a dose of 25 mg/week compared to a placebo in a 1:1 ratio among 100 recently diagnosed PMR patients, in accordance with the 2012 EULAR/ACR criteria. All participants will receive prednisolone at a dose of 15 mg/day, which will be tapered to 0 mg over 24 weeks. In cases of primary non-response or disease relapse, the prednisolone dose will be allowed to be temporarily increased. Assessments will occur at baseline, as well as at 4, 12, 24, 32, and 52 weeks. The primary outcome measure will be the difference in the proportion of patients achieving glucocorticoid-free remission by week 52 [73]. Methotrexate and leflunomide are also being examined in the STERLING-PMR study, targeting patients with PMR who have experienced at least one relapse (ISRCTN17828080). This phase III trial is an open-labelled, randomized controlled study, planning to include up to 200 patients, that compares standard care versus standard care plus methotrexate or leflunomide if methotrexate is not tolerated. Methotrexate will be started at 15 mg weekly and progressively increased to 25 mg or reduced to 10 mg weekly if not tolerated. If 10 mg weekly methotrexate is not tolerated, or not effective, it may be switched to leflunomide starting at 10 mg daily and then increased to 20 mg daily if tolerated or reduced to 10 mg every 2 days if not tolerated.

Alternatives like azathioprine show limited efficacy, with small studies supporting their use but raising concerns about side effects [75]. It may also be the case for hydroxychloroquine in PMR patients when compared to glucocorticoids alone [76].

#### 3.2.2. Biologic Therapies in PMR

##### Anti-TNF Therapy

**Figure 1:**
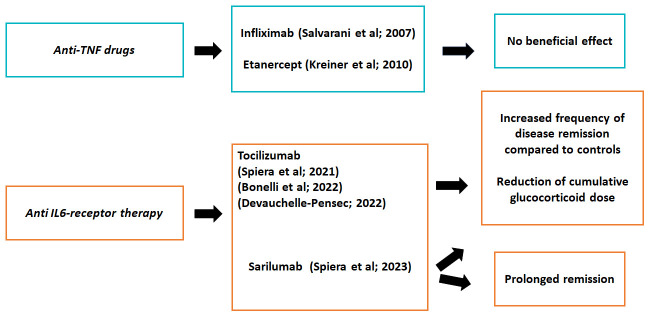
Randomized controlled clinical trials in isolated polymyalgia rheumatica (PMR) patients undergoing tumor necrosis factor (TNF)α or interleukin (IL6)-receptor antagonist therapies [78,80,81,82,83,84].

Initial studies on tumor necrosis factor (TNF)-α antagonists in PMR, mainly based on single or small series revealed promising results [77]. However, the only randomized clinical trial on the efficacy of infliximab in PMR did not disclose an additional benefit of adding this monoclonal antibody to prednisone to treat newly diagnosed patients with PMR [78] (Figure 1). This study assessed the efficacy of infliximab in PMR patients with newly diagnosed disease, specifically those who were corticosteroid naïve. The study included 51 patients with a mean age of 71 years and compared infliximab (3 mg/kg intravenous (I.V.)) to a placebo, both in conjunction with a tapering of the prednisone dose starting at 15 mg/day. At 52-week follow-up, the primary endpoint (the proportion of patients free of relapses) showed no significant difference between the two groups. In addition, there were no significant differences in the number of relapses, their duration, or the cumulative dose of corticosteroids [78]. Concerns were raised regarding the small sample size and rapid tapering of glucocorticoids, which could have influenced the results. These limitations have been echoed by experts in the field, highlighting the need for caution when interpreting the results [79].

Another randomized controlled trial with etanercept in monotherapy versus a placebo in patients with PMR did not meet primary or main secondary end points either [80]. Considering these results, the 2015 EULAR/ACR recommendations did not support the use of anti-TNF agents for the management of isolated PMR [56].

##### Anti-Interleukin-6 Therapy

IL-6 is key proinflammatory cytokine that plays an important role in the pathogenesis of PMR [85,86]. A reduction in serum IL-6 levels has been linked to decreased disease activity, making IL-6 blockade a potential therapeutic option for PMR, similar to its use in GCA [87].

The first studies were performed using tocilizumab, a monoclonal antibody that competitively inhibits IL-6 binding to its receptor. By blocking the entire receptor complex, it prevents IL-6 signal transduction to inflammatory mediators involved in B- and T-cell activation [88].

As previously mentioned, GCA and PMR are overlapping and frequently concurrent diseases [89]. The beneficial effect of the anti-IL-6 receptor tocilizumab in GCA patients, many of them with PMR manifestations, reported in observational studies [90,91], was corroborated in two prospective clinical trials [92,93]. Since tocilizumab produced rapid improvement of PMR symptoms in patients with GCA, the use of anti-IL-6 receptor agents to control relapsing or refractory PMR became a plausible approach. In this regard, several reports, some of them describing single cases [94,95,96] and others including between two and three patients with PMR showed that tocilizumab yielded improvement of PMR manifestations without relapses leading to remission of the disease [97,98,99]. In keeping with the findings of these case reports, Toussirot et al. performed a retrospective evaluation of seven patients with PMR, six of whom had isolated “pure” PMR and one with asymptomatic biopsy-proven GCA. Five of the patients were glucocorticoidrefractory, requiring a median prednisone dose of 20 mg/day, while the other two had diabetes and metabolic syndrome features. All patients were treated with I.V. tocilizumab at 8 mg/kg/month as monotherapy. Within 4 to 8 weeks, all patients showed rapid clinical improvement, with reductions in both PMR activity scores and CRP levels. Half of the patients achieved low disease activity, and disease remission was obtained in three patients, with no further PMR relapses [100].

A larger study was conducted by Assaraf et al. [101], who performed a multicenter retrospective observational study involving fifty-three patients with PMR, thirty-one of whom had persistent symptoms of PMR despite treatment with conventional, synthetic, disease-modifying antirheumatic drugs. As in the former study, they were also treated with monthly I.V. tocilizumab therapy. Results of this study showed that, in PMR patients who are glucocorticoid dependent, the use of anti-IL-6 receptor tocilizumab therapy led to a significant reduction in the dose of glucocorticoids, leading to disease remission.

Of greater importance was the confirmation of the beneficial effects of anti-IL-6 receptor therapy through prospective studies (Figure 1). To evaluate the effects of a short initial course of tocilizumab as a first-line treatment without glucocorticoids, Devauchelle-Pensec et al. conducted a prospective, open-label study. The study included 20 patients with newly diagnosed active PMR, who were administered three infusions of tocilizumab at a four-week interval. After 12 weeks of follow-up, patients showed clinical improvement of PMR symptoms [102]. To further explore the efficacy of glucocorticoid-free tocilizumab monotherapy in the treatment of PMR, Chino et al. conducted a 2-year, prospective, single-center, open-label pilot study involving thirteen patients. Participants received I.V. tocilizumab at a dose of 8 mg/kg every 2 weeks for the first 2 months, followed by monthly infusions for the next 10 months [103]. After completing the treatment phase, patients were followed for another year without any treatment. Primary endpoints were remission rates at weeks 12 and 52, while secondary endpoints included relapse rates and safety over the 104-week study period. At week 12, four patients achieved remission, although four others withdrew due to adverse events or lack of efficacy. At week 52, all nine patients who completed the first year were in remission. Of the eight patients who completed the second year without drug, seven maintained remission. The study demonstrated the beneficial effects of tocilizumab in the management of PMR without the need for glucocorticoids [103].

In a separate study, 10 patients with newly diagnosed PMR participated in a prospective, open-label, single-center phase IIa trial of tocilizumab. Patients were enrolled within one month of their PMR diagnosis and were required to have initially received no more than 20 mg/day of prednisone, or its equivalent, to be included in the study. Patients received I.V. tocilizumab (8 mg/kg/month) for one year, along with rapid glucocorticoid tapering following a standardized protocol. In this regard, following the initial infusion of tocilizumab, glucocorticoid daily dose tapered by 2.5 mg every 2 weeks [104]. A control group included patients with PMR who refused to take tocilizumab or did not meet inclusion criteria. Of note, nine of the 10 patients treated with tocilizumab achieved relapse-free, glucocorticoid-free remission at 6 months. Furthermore, they were able to discontinue glucocorticoid therapy within 4 months of starting the trial, maintaining remission over the 15-month study period. In contrast, none of the patients in the control group achieved glucocorticoid-free remission at 6 or 12 months, and 60% experienced relapses. Furthermore, the median cumulative prednisone dose was significantly lower in the tocilizumab-treated group (1085 mg) compared with the control group (2562 mg). These findings provided support for the use of the anti-IL6 receptor tocilizumab as a glucocorticoid-sparing agent in patients with active PMR [104].

Additional information was obtained from randomized controlled trials. One of them was a post hoc analysis of the Giant-Cell Arteritis Actemra (GiACTA) trial [81]. It included fifty-two patients with newly diagnosed or recurrent isolated PMR. PMR patients were treated with subcutaneous tocilizumab (162 mg weekly or every other week) plus a tapering glucocorticoid regimen for 26 weeks (thirty-one patients) or a placebo combined with a tapering glucocorticoid regimen for 26 or 52 weeks (twenty-one patients). The duration of the study was 52 weeks. Remission, defined as no relapses and CRP levels below 1 mg/dL at week 52, was maintained in 45% of the tocilizumab group, compared with 19% in the placebo group. The median glucocorticoid dose at week 52 was significantly lower in the tocilizumab group (1862 mg) versus the placebo group (3671 mg). Serious adverse events occurred in 16% of the tocilizumab group and 14% of the placebo group [81].

To assess the efficacy of anti-IL-6 receptor tocilizumab in patients with new-onset PMR, Bonelli et al. conducted a phase 2/3 randomized controlled trial. The study randomly assigned thirty-six patients with new-onset PMR from three centers to receive subcutaneous tocilizumab (162 mg weekly) or a placebo for 16 weeks, in a 1:1 ratio. All patients also received oral prednisone, the dose of which was reduced from 20 mg to 0 mg over 11 weeks. Thus, the experimental group received tocilizumab along with a glucocorticoid tapering regimen, whereas the control group received a placebo with glucocorticoids. Tocilizumab was found to be effective in reducing glucocorticoid use and resulted in fewer side effects compared with traditional treatments [82].

Another study conducted by Devauchelle-Pensec et al. evaluated the impact of tocilizumab on disease activity in patients with active PMR, particularly that undergoing glucocorticoid therapy [83]. This double-blind, parallel-group, placebo-controlled randomized clinical trial enrolled one hundred and one patients with PMR from 17 French hospitals. Inclusion criteria required patients to have persistent disease activity, indicated by the CRP-PMR activity score (CRP-PMR-AS), which is a PMR activity score calculated using CRP greater than 10 (range of the score 0–100) and a prednisone dose of at least 10 mg per day. Participants were randomly assigned to receive either I.V. tocilizumab (8 mg/kg; fifty-one patients) or a placebo (fifty patients) every four weeks for 24 weeks, alongside a standardized tapering regimen of oral prednisone. The primary efficacy end point was CRP-PMR-AS less than 10 (range, 0–100; higher values indicate greater activity) combined with either prednisone dose less than or equal to 5 mg per day or a decrease in prednisone dose greater than or equal to 10 mg from baseline at week 24. The study demonstrated that tocilizumab significantly reduced disease activity in patients with PMR compared to the placebo, with those receiving tocilizumab showing greater improvements in symptoms and a decreased need for glucocorticoids. Additionally, tocilizumab was well tolerated, exhibiting a safety profile comparable to that of other treatments for inflammatory conditions. The findings of this interesting study support the potential use of tocilizumab to enhance management strategies for PMR, particularly for patients who experience glucocorticoid-related side effects [83].

Unlike previous studies that examined the role of tocilizumab in PMR, Spiera et al. assessed the efficacy of sarilumab, another IL-6 receptor antagonist, for managing relapses of PMR during glucocorticoid tapering [84]. A randomized controlled trial was conducted to compare sarilumab versus a placebo. In this phase 3 trial, patients were randomly assigned in a 1:1 ratio to receive either sarilumab (200 mg via subcutaneous injection every two weeks for 52 weeks, along with a 14-week prednisone taper) or a placebo with a 52-week prednisone taper.

Sixty patients received sarilumab, and fifty-eight received the placebo. The primary outcome at 52 weeks was sustained remission, defined as the resolution of PMR signs and symptoms by week 12, sustained normalization of CRP levels, absence of disease flare, and adherence to the prednisone taper from weeks 12 through 52. At week 52, sustained remission was significantly more frequent in the sarilumab group (28% vs. 10% in the placebo group). Additionally, at 52 weeks, patients treated with sarilumab had a significantly lower median cumulative glucocorticoid dose (777 mg vs. 2044 mg in the placebo group) [84].

Neutropenia was more common in the sarilumab group (15% vs. 0%), as were arthralgia (15% vs. 5%) and diarrhea (12% vs. 2%). Treatment-related discontinuations were also more frequent in the sarilumab group than in the placebo group (12% vs. 7%).

The results of this study demonstrated that sarilumab significantly reduces the rate of relapse during glucocorticoid tapering, indicating its potential as a viable treatment option for patients experiencing this common complication of PMR. This study is clinically relevant, as PMR often requires prolonged glucocorticoid therapy, which can lead to significant side effects. Therefore, it addressed a crucial need for effective management strategies during tapering periods, making it highly relevant for both clinicians and patients [84]. Subsequently, the FDA-approved sarilumab as the first biologic agent indicated for patients with PMR.

##### Exploring Additional Biologic Agents

Information on the potential benefits of other biologic agents for patients with isolated PMR is limited. Interleukin-1 (IL-1) and IL-17 are two of the major proinflammatory cytokines implicated in disease pathogenesis [105]. As a result, the human IgGκ monoclonal antibody canakinumab, which targets IL-1β, and secukinumab, which specifically targets IL-17A, were investigated in patients with isolated PMR.

In this regard, Matteson et al. conducted a two-week, single-blind, randomized proof-of-concept trial to assess the effects of secukinumab, canakinumab, and glucocorticoids on disease activity in patients with PMR. The study included three treatment groups, in which participants received secukinumab, canakinumab, or glucocorticoids [106]. The primary objective was to assess changes in disease activity scores following treatments over the two-week period. After this phase, an open-label extension provided continued treatment to participants, offering additional information on the long-term effects and tolerability of the biologic agents. The results showed that while patients receiving glucocorticoids experienced rapid pain relief, those receiving biologic treatments showed only moderate improvements in mobility. At the end of the two weeks, none of the patients receiving biologic agents achieved a complete response, emphasizing the need for further research into the efficacy of these agents in the treatment of PMR [106].

The REPLENISH study (NCT05767034) is a phase III, randomized, double-blind, placebo-controlled trial aimed at evaluating the efficacy and safety of secukinumab in patients with glucocorticoid-dependent polymyalgia rheumatica (PMR) experiencing a relapse. The trial involves administering 150 mg or 300 mg of secukinumab every 4 weeks, alongside a tapering glucocorticoid regimen over 24 weeks. The goal is to assess secukinumab’s potential in reducing the reliance on long-term glucocorticoids, which can have serious side effects. The trial is expected to conclude by February 2026 and is recruiting around 360 participants.

Rituximab was evaluated in a randomized trial suggesting its efficacy in recent-onset PMR. The study included 47 patients, 38 of whom had recently been diagnosed with PMR [107]. A single dose of 1000 mg rituximab or placebo was administered, along with prednisone (15 mg/day) with a rapid taper in 4 months. The primary endpoint was focused on disease activity using the PMR AS score (range 0–100) that had to be of less than or equal to 10 at week 21. This endpoint was achieved by 48% of patients in the rituximab group versus 21% in the placebo group. This significant difference observed at week 21 was no longer significant at one year [108]. Therefore, there is currently insufficient robust clinical evidence to definitively determine whether rituximab is effective in treating PMR. In this regard, there are two ongoing phase III, randomized, double-blind, placebo-controlled studies designed to assess the effectiveness of rituximab in PMR, REDUCE-PMR-1 and REDUCE-PMR-2 trials (NCT05533125, NCT05533164). REDUCE-PMR-1 is focused on newly diagnosed PMR patients. Participants receive a single 1000 mg intravenous dose of rituximab, along with a 17-week glucocorticoid tapering protocol starting with 15 mg/day prednisolone. The trial aims to assess rituximab’s ability to promote remission while minimizing glucocorticoid use. If a relapse occurs, an additional lower dose of rituximab may be given at week 24. In contrast, the REDUCE-PMR-2 focuses on patients experiencing a relapse of PMR. The design and objectives are similar, with rituximab tested against a placebo in conjunction with a glucocorticoid taper.

Abatacept, a dimeric fusion protein that blocks the interaction between CD80/CD86 and CD28, thereby reducing T-cell activation, has been used in the treatment of GCA [109]. Abatacept was assessed in patients with early PMR in an attempt to determine the efficacy of this biologic agent to achieve low disease activity without glucocorticoids. For this purpose, Saraux et al. performed a proof-of-concept, randomized, double-blind, placebo-controlled, parallel-group trial that included thirty-four PMR recited in five French center. Patients included in this study were required to have a disease duration of less than 6 months and a PMR-CRP activity score (PMR-AS) greater than 17 with no signs or symptoms of GCA confirmed by clinical evaluation and PET-CT evaluation [110]. Participants were randomly assigned (1:1) to receive weekly subcutaneous abatacept (125 mg) or a matching placebo, with glucocorticoid rescue therapy allowed in cases of high disease activity, for 12 weeks, and then glucocorticoid treatment based on disease activity, until week 36. The primary endpoint was low disease activity (CRP PMR-AS ≤ 10) at week 12 without glucocorticoids and without rescue treatment. The primary endpoint was reached by eight of sixteen patients in the abatacept group and four of eighteen patients in the placebo group. Eight patients in the abatacept had adverse events. Although PMR treated with abatacept experienced significant improvements in disease activity compared to the placebo group, the interpretation raised as the final conclusion of this study suggested that the effect of abatacept alone is not strong enough to justify larger studies in early PMR [110].

Also, a phase II, open-label, single-center trial (NCT04062006) is investigating the use of low-dose Interleukin-2 (IL-2) in PMR patients. This study aims to evaluate both the clinical and immunological effects of administering low-dose IL-2. Patients receive subcutaneous injections of recombinant human IL-2 at a dose of 1 million units, five days per week for the first 4 weeks, followed by weekly doses for an additional 8 weeks. The study follows up with patients for 3 months post-treatment to assess changes in clinical and laboratory markers, as well as immune cell subsets and cytokine levels. The trial targets patients aged 50 or older with stable glucocorticoid regimens and without significant comorbidities, such as serious infections or cancer. The goal is to determine whether IL-2 can help reduce disease activity and glucocorticoid dependence in PMR patients.

##### Role of Targeted, Synthetic, Disease-Modifying Anti-Rheumatic Drugs

The Janus kinase/signal transducers and activators of transcription (JAK/STAT) pathway plays a crucial role in cellular regulation in humans. This pathway is utilized by a wide range of cytokines involved in the pathogenesis of autoimmune diseases to transmit intracellular signals. Additionally, various polymorphisms in JAK and STAT genes have been linked to autoimmune conditions. Notably, elevated levels of interferon-gamma mRNA have been observed in the temporal arteries of patients with GCA who experience severe ischemic complications. The JAK/STAT inhibitor tofacitinib, which targets JAK3 and JAK1, has been shown to prevent T-cell accumulation in the vessel wall and to suppress IFN-γ production and signaling through this pathway [111,112].

Ma et al. evaluated the efficacy of tofacitinib in patients with PMR. They found marked increases in the expression of several key inflammatory markers including IL6R, IL1B, IL1R1, JAK2, Toll-like receptors (TLR2, TLR4, and TLR8), C-C chemokine receptor type 1 (CCR1), complement receptor 1 (CR1), and calgranulins S100A8 and S100A12 in 11 newly diagnosed patients with PMR [113]. In vitro studies demonstrated that tofacitinib effectively suppressed the expression of IL-6R and JAK2 in CD4+ T-cells from these patients. They subsequently randomly assigned treatment-naïve PMR patients to receive either tofacitinib or glucocorticoids for 24 weeks, with assessments performed at multiple time points (0, 4, 8, 12, 16, 20, and 24 weeks) to calculate PMR activity disease scores (PMR-ASs). The primary endpoint was the proportion of patients achieving a PMR-AS of ≤10 at weeks 12 and 24, while secondary endpoints included changes in PMR-ASs, CRP, and the ESR. Thirty-nine patients were treated with tofacitinib, and thirty-seven received glucocorticoids, and thirty-five and thirty-two patients completed the study, respectively. However, no significant differences were observed in the primary or secondary outcomes. At weeks 12 and 24, all patients in both groups achieved PMR-ASs of below 10, with significant reductions in the PMR-AS, CRP, and ESR in both treatment groups. Importantly, no serious adverse events were reported. However, the study found no statistically significant differences between tofacitinib and glucocorticoids in the treatment of PMR [113].

The BACHELOR study was a phase II, multicenter, double-blind, randomized placebo-controlled trial that explored the efficacy of baricitinib, a JAK1/2 inhibitor, in early PMR without glucocorticoids. The study included patients who had not taken glucocorticoids in the prior two weeks, and they were randomized to receive either 4 mg of baricitinib daily or a placebo for 12 weeks. If remission was achieved (PMR-AS ≤ 10), the dose was reduced to 2 mg daily for an additional 12 weeks. The primary goal was to assess the proportion of patients achieving sustained low disease activity without needing glucocorticoids. Glucocorticoid rescue was allowed at the discretion of the investigator for high disease activity. Results showed that by week 12, a significantly higher proportion of patients in the baricitinib group (77.8%) reached the primary endpoint compared to the placebo group (13.3%). Additionally, patients receiving baricitinib had better health-related quality of life scores and experienced fewer adverse events. This suggests that baricitinib could be effective in managing early PMR without relying on glucocorticoids. However, full results have not yet been published.

The JAK-SPARE1 phase III study is currently recruiting to further assess baricitinib’s efficacy in achieving glucocorticoid-free remission in newly diagnosed PMR patients after 16 weeks of treatment.

## 4. Discussion

PMR is an inflammatory condition prevalent among people over 50 years of age of European ancestry [1,2,4,14,41]. Glucocorticoids are the primary treatment for PMR [51,56]. Although an initial dose of 12.5 to 25 mg/day of prednisone/prednisolone may result in rapid relief of symptoms, relapses are common, especially during medication tapering [30]. This often results in prolonged use of glucocorticoids, which can cause adverse side effects such as diabetes, osteoporosis and vertebral fractures, femoral neck fractures, hip fractures, cardiovascular disease, and ocular comorbidity, ultimately undermining outcomes for patients with this condition [114,115]. As a result, management strategies incorporating both conventional and biological disease-modifying anti-rheumatic agents have been implemented to induce disease remission, decrease the frequency of relapses, and reduce the cumulative prednisone dose, which contributes to comorbidities in PMR patients. Traditionally, the 2015 EULAR/ACR guidelines suggest considering early use of methotrexate for patients at high risk of relapses or those requiring prolonged therapy, particularly if they have comorbidities or are on medications that heighten the risk of glucocorticoid side effects [51,56]. Although the use of methotrexate and leflunomide in PMR has been reported to reduce the frequency of disease relapses and promote remission in many cases [66,67,68,69,70,71], the findings of these studies do not consistently support their effectiveness across all investigations [68]. Consequently, more research is needed to better evaluate the impact of methotrexate and leflunomide on PMR [72].

Biologic agents are employed in PMR patients who have refractory disease, experience relapses, or require a rapid discontinuation of glucocorticoids due to comorbidities that render their use undesirable. However, randomized controlled trials do not support the effectiveness of anti-TNF agents in the management of PMR [78,80].

In contrast, in keeping with the positive findings reported in retrospective studies [57,79], several placebo-controlled trials (three with tocilizumab and one with sarilumab) support the beneficial effect of the anti-IL-6 receptor blockade for the management of PMR [81,82,83,84]. Three studies utilized tocilizumab, a monoclonal antibody that specifically targets the IL-6 receptor, thereby preventing IL-6 from binding to it [81,82,83]. A similar mechanism of action has, sarilumab, another human monoclonal antibody that acts against the IL-6 receptor [84]. In this regard, the use of anti-IL6 receptor agents was linked to rapid improvement of pain and stiffness in patients with PMR. Additionally, these agents facilitate a reduction in the cumulative glucocorticoid dosage, thereby lowering the risk of side effects associated with long-term glucocorticoid therapy. Furthermore, data from these studies suggest that PMR patients receiving anti-IL-6 receptor therapy may experience a reduced rate of disease relapses compared to those treated with glucocorticoid in monotherapy [81,82,83,84]. As a result, patients often experience enhanced quality of life due to an improved functional status and reduced disease burden. Currently, the only biologic agent approved by the FDA specifically for the treatment of PMR is sarilumab, which is administered subcutaneously every two weeks. This biologic agent has a favorable safety profile and can provide sustained remission, making it especially beneficial for patients who have difficulty maintaining disease remission with traditional therapies [84].

The use of secukinumab, canakinumab, rituximab, and abatacept in PMR is an area of ongoing research, with varying degrees of evidence and clinical experience [106,107,108,109,110]. However, more research is needed to determine the efficacy of these biologic agents in patients with isolated PMR. This is also the case for the use of targeted synthetic disease-modifying anti-rheumatic dugs in the management of isolated PMR and other inflammatory or autoimmune diseases [111,112]. In this regard, although in vitro studies showed that the JAK1/JAK3 inhibitor tofacitinib effectively suppressed the expression of IL-6R and JAK2 in CD4+ T-cells from patients with PMR, no significant difference in disease activity scores was observed between patients with PMR treated with tofacitinib and those treated with glucocorticoids [113]. Therefore, the potential favorable effects of JAK inhibitors in isolated PMR remain to be determined.

## 5. Conclusions

Glucocorticoids (such as prednisone or prednisolone) remain the primary treatment for PMR, as they help reduce inflammation and control symptoms such as pain and stiffness. However, long-term use of glucocorticoids is known to cause several side effects, including osteoporosis, diabetes, and weight gain. These risks are even greater for patients who already had other health conditions before starting glucocorticoids and for those who experience frequent relapses when the glucocorticoid dose is reduced or stopped.

To address this, glucocorticoid-sparing agents are being explored as alternatives. Among these, methotrexate (a conventional disease-modifying drug) and biologic agents, particularly anti-IL-6 therapies, such as tocilizumab and sarilumab, have shown promise. These biologics target the IL-6 pathway, which plays a key role in PMR-related inflammation. Studies have shown that blocking IL-6 receptors results in rapid relief of pain and stiffness, making it easier to reduce glucocorticoid doses and decreasing the risk of long-term glucocorticoid side effects. This can help achieve sustained remission in many patients.

However, while biologics can be effective, they come with considerations such as a higher risk of side effects and significantly higher costs compared to traditional treatments such as glucocorticoids and methotrexate. Therefore, the decision to use biologics should carefully weigh the benefits and potential risks for each patient.

**Figure 2:**
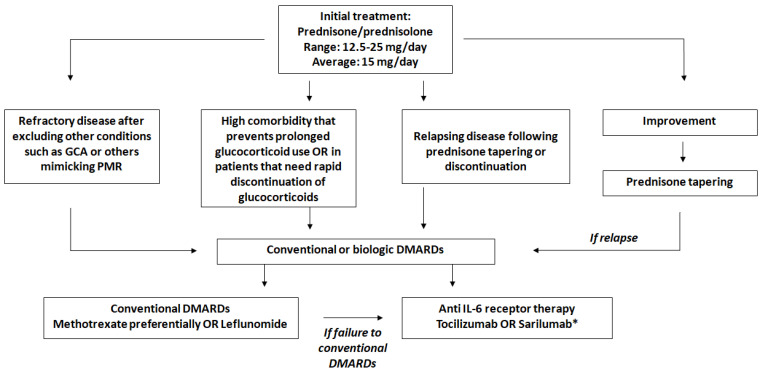
Proposed management of patients with isolated PMR. *: Approved by the FDA for PMR. Abbreviations: GCA = Giant Cell Arteritis; PMR=Polymyalgia Rheumatica; DMARDs = disease modifying anti-rheumatic drugs; IL6 = interleukin 6; TNF = tumor necrosis factor.

Taking into account all the considerations and evidence described in this review, Figure 2 illustrates our proposed approach to managing patients with isolated PMR.

## 6. Future Directions

As experts in the field have recently highlighted, it is important to establish specific recommendations in the treatment of patients with PMR [114]. This approach includes achieving and maintaining disease remission and preventing complications.

Treatment should prioritize shared decision-making between patients and physicians, and comorbidities should be taken into account in treatment planning. In this regard, individualized care is important, with glucocorticoids being a key treatment in PMR, but considering the possibility of using glucocorticoid-sparing therapies such as biological agents [114].

The arrival of biosimilars of tocilizumab, and later of sarilumab, may facilitate earlier use of these agents, as they are more cost-effective. Therefore, it is crucial to optimize referral, diagnosis, and management strategies to minimize glucocorticoid dependence while achieving remission, which is the primary goal of treatment [115]. Accurate diagnosis and early stratification of patients with PMR, excluding conditions such as underlying large vessel extracranial GCA in patients presenting with PMR, is of major importance [116,117].

Optimizing the benefit/risk ratio of glucocorticoids to minimize adverse events while achieving sustained remission remains an ongoing challenge. Therefore, further research on alternative therapies for glucocorticoid treatment in PMR will be crucial to improve patients’ quality of life and reduce comorbidities associated with long-term glucocorticoid use. In this regard, obesity is associated with poorer outcomes for various patient-reported outcome measures in individuals with PMR. Therefore, weight management should be considered when treating patients with PMR, especially in obese individuals [118].

The development of innovative glucocorticoid preparations and glucocorticoid receptor ligands may enhance the benefit/risk ratio of glucocorticoids, reducing side effects while maintaining their therapeutic efficacy. In this regard, one approach might be the use of selective Glucocorticoid Receptor Agonists and Modulators (SEGRMs) that are designed to selectively activate anti-inflammatory pathways without triggering the pathways responsible for glucocorticoid-related side effects. This selective activation allows SEGRMs to minimize common glucocorticoid-related adverse effects, such as osteoporosis, diabetes, and cardiovascular issues, while still exerting their potent anti-inflammatory effects in patients with PMR [119,120].

Another potential emerging therapy may be the use of liposome-based drug delivery systems, which are small, nanometer-sized particles, which have been explored in the treatment of RA [121,122]. These liposomes can encapsulate glucocorticoids and deliver them selectively to inflamed tissues, increasing local efficacy while minimizing systemic exposure and reducing side effects. Research suggests that this technology could potentially be applied to other inflammatory diseases, such as GCA and PMR, in the future.

The potential utility of immuno-checkpoints in managing PMR is another emerging area of research. Commonly used in cancer therapy, this promising line of research has not yet been extensively explored in PMR. However, there is potential for trials to evaluate their efficacy in managing inflammation and achieving remission in PMR. In this regard, combining immuno-checkpoint inhibitors with existing treatments such as glucocorticoids or biologic agent could enhance therapeutic outcomes and minimize glucocorticoid exposure, reducing the risk of associated side effects [123]. This may be of great interest in particular for the management of PMR patients with pre-existing comorbidities [124,125].

However, research on these new approaches, such as SEGRMs and immune checkpoints, is still in early stages of investigation, with no conclusive results regarding their effectiveness in treating PMR.

Further research is needed to fully establish their efficacy and broader applicability.
